# The relationship between muscle sympathetic nerve activity and serum fatty acid binding protein 4 at rest and during isometric handgrip exercise

**DOI:** 10.14814/phy2.70122

**Published:** 2024-12-26

**Authors:** Tadayuki Hirai, Takuto Hamaoka, Hisayoshi Murai, Hiroyuki Sugimoto, Yusuke Mukai, Ayano Nomura, Takashi Kusayama, Tatsunori Ikeda, Shinichiro Takashima, Takeshi Kato, Kenji Sakata, Soichiro Usui, Shigeo Takata, Masayuki Takamura

**Affiliations:** ^1^ Department of Cardiovascular Medicine Kanazawa University Graduate School of Medical Sciences Kanazawa Japan; ^2^ Heart and Vascular Institute Pennsylvania State University College of Medicine Hershey Pennsylvania USA; ^3^ Kanazawa Municipal Hospital Kanazawa Japan

**Keywords:** catecholamines, FABP4, handgrip exercise, lipolysisv, sympathetic nervous system

## Abstract

Fatty acid binding protein 4 (FABP4) is highly expressed in adipocytes. Lipolysis, caused by an elevated adrenergic input, has been suggested to contribute to elevated serum FABP4 levels in patients with cardiovascular diseases. However, the relationship between the serum FABP4 and efferent sympathetic nerve activity remains poorly understood. Twenty‐one healthy subjects (average age, 29.1 years; 15 men) performed an isometric handgrip (HG) exercise at 30% of the maximal voluntary contraction until they were fatigued. The beat‐by‐beat heart rate (HR), blood pressure (BP), and muscle sympathetic nerve activity (MSNA) were recorded. Blood samples were collected at rest and at the time of peak fatigue. The MSNA, HR, and systolic BP were significantly increased by the HG exercise (all, *p* < 0.05). MSNA was obtained from 14 patients. The change in the FABP4 on HG exercise was significantly correlated with the change in MSNA (bursts/100 heartbeats) (*R* = 0.808, *p* < 0.001) but not with changes in other parameters, which might, in part, reflect an association of efferent sympathetic drive with FABP4. Meanwhile, resting FABP4 levels were not associated with any parameters including MSNA, in healthy individuals. Future studies on patients with elevated sympathetic activity are warranted to examine the relationship further.

## 1 INTRODUCTION

Fatty‐acid‐binding proteins play crucial roles in several metabolic and inflammatory pathways, principally via transport of fatty acids to specific organelles within cells (Coe & Bernlohr, 1998). Fatty acid binding protein 4 (FABP4), also known as adipocyte‐FABP, is commonly expressed in adipose tissue (Spiegelman et al., 1983), and regulates lipid metabolism and inflammation (Hunt et al., 1986). Serum FABP4 has been regarded as a biomarker of adiposity because of the significant relationship between levels thereof and lipid profiles (e.g., high LDL and triglycerides, and low HDL) (Cabré et al., 2007; Furuhashi et al., 2016; Hotamisligil & Bernlohr, 2015). Serum FABP4 levels predicted the development of metabolic syndrome and type 2 diabetes (Chen et al., 2017). Also, FABP4 concentration is associated with both the development and severity of cardiovascular diseases (CVDs) including hypertension (Chen et al., 2017), coronary artery disease (Kralisch & Fasshauer, 2013), ischemic stroke (Hansen et al., 2021), and fatal arrhythmia (Wang et al., 2019). Notably, the FABP4 level predicted future CVDs independent of traditional risk factors including obesity and dyslipidemia (Chow et al., 2013). Therefore, it is speculated that FABP4 is not just a surrogate measure of adiposity.

In an animal model, lipolysis increased FABP4 expression (Ertunc et al., 2015). Lipolysis is commonly induced by catecholamines via sympathetic nerve activation (Rodríguez‐Calvo et al., 2017). Therefore, it seemed possible that such activation would increase the serum FABP4 level, predisposing subjects to CVDs/events (Lee et al., 2021). An earlier report found a significant correlation between the serum norepinephrine (NE) and FABP4 concentrations of healthy humans during exercise (Iso et al., 2017). Although serum NE is a well‐known product of sympathetic activation, it is not an optimal marker of sympathetic nerve activity (SNA) (Armario et al., 2020). Also, the prior study utilized highly intensive exercise (e.g., loads that reached the anaerobic threshold using an ergometer) (Numao et al., 2023, 2024), yet the FABP response to relatively lower‐intensity exercise is still unclear. Muscle sympathetic nerve activity (MSNA) is an established marker of human efferent SNA innervating peripheral vascular bed (White et al., 2015). MSNA has been used in studies evaluating SNA at rest and during physiological stress (D'Souza et al., 2023); an augmented MSNA was associated with CVD severity and prognosis (e.g., of heart failure) (Mukai et al., 2002; Murai et al., 2009). However, any relationship between MSNA and the serum FABP4 level remains unknown. Thus, this study investigated the relationship between MSNA and the FABP4 level in healthy individuals at rest and during handgrip (HG) exercise. We hypothesized that the FABP4 level at rest and the change by isometric HG exercise would be correlated with MSNA.

## 2 METHODS

### 2.1 Participants

Twenty‐one young healthy subjects (male/female, 15/6; average age, 29.1 ± 4.7 years) were consecutively enrolled (Table 1). Their health status and medical history were checked using a questionnaire. All participants had received annual medical check‐ups and no disease had been detected. The study protocol was approved by the Research Ethics Board of Kanazawa University (Kanazawa, Japan) and conformed with the Declaration of Helsinki. The study has been registered in the University Hospital Medical Information Network Center (Tokyo, Japan) Clinical Trials Registration System (no. UMIN000039755). All participants provided written informed consent. The datasets generated during and/or analyzed during the current study are not publicly available but may be obtained from the corresponding author on reasonable request.

**TABLE 1:** Baseline characteristics.

Subjects (male/female)	21 (15/6)
Age (years)	29.1 ± 4.7
Height (m)	1.68 ± 0.1
Body weight (kg)	62.0 ± 11.6
Body mass index (kg/m^2^)	21.7 ± 3.0
Heart rate (bpm)	64 ± 9
Systolic blood pressure (mmHg)	118 ± 11
Creatinine (mg/dL)	0.73 ± 0.15
eGFR (ml/mi/1.73m^2^)	99.98 ± 18.17
Glucose (mg/dL)	95.2 ± 18.8
Insulin (μU/mL)	13.7 ± 13.0
HbA1c (%)	5.0 ± 0.2
Triglycerides (mg/dL)	85.4 ± 46.4
Norepinephrine (pg/mL)	202.1 ± 81.0
FABP4 (ng/mL)	14.3 ± 5.3

*Note*: The values are numbers of subjects or means ± SDs.

Abbreviations: eGFR, estimated glomerular filtration rate; FABP4, fatty acid binding protein 4; HbA1c, glycosylated A1a‐1 hemoglobin.

### 2.2 Measurements

The beat‐by‐beat blood pressure (BP) of the radial artery was non‐invasively and continuously recorded using a tonometry system (JENTOW‐7700; Nihon Colin, Komaki, Japan) (Sato et al., 1993). Heart rate (HR) was determined via continuous electrocardiographic recording. The MSNA of the right peroneal nerve was measured using a previously described microneurographic technique (Delius et al., 1972; Sugimoto et al., 2022; Vallbo et al., 1979). Briefly, a tungsten microelectrode was percutaneously inserted into the peroneal nerve and a reference electrode was positioned subcutaneously 1–3 cm from the recording site. The electrode was connected to a preamplifier operating at a gain of 1000 and then to an amplifier with a gain of 70. The signals were band‐pass filtered (500–3000 kHz) using the resistance‐capacitance integrated circuit with a time constant of 0.1 s of a PowerLab recording system (Model ML 785/85P; ADI Instruments, Bella Vista, NSW, Australia). The raw nerve signal was obtained at 12 kHz. Other signals were obtained at 1000 Hz. The electrode was adjusted until MSNA bursts were detected. An MSNA burst was identified by the characteristic pulse‐synchronous pattern that increased in response to voluntary apnea, the absence of skin paresthesia, and no response during arousal to loud noise. Once offline, these bursts were further evaluated using LabChart software (v.8; ADInstruments). Fixed criteria, including an appropriate latency after the R‐wave of the electrocardiogram were applied to recognize the MSNA burst (Delius et al., 1972). MSNA bursts were expressed as numbers/min (burst frequency, BF) (bursts/min) and numbers per 100 heartbeats (burst incidence, BI) (bursts/100 heartbeats).

### 2.3 Experimental protocol

All subjects were asked to abstain from alcohol, caffeine, and strenuous exercise for 24 h before the study visit. The subjects were requested to take a light meal in the morning to avoid hunger and satiety. Measurements were performed between 10:00 and 12:00 a.m. in a dimly lit room at a comfortable temperature (∼23°C). An intravenous catheter was inserted into the antecubital vein of the non‐dominant arm to draw blood. At study commencement, each subject performed three HG exercises (using the non‐dominant arm) employing a HG dynamometer (Smedley Hand Dynamometer; Toei Light, Tokyo, Japan) and the largest grip strength was defined as the maximal voluntary contraction (MVC). Before the actual recording, instruction of HG exercise and practice opportunity were provided. The participants were supine during MVC measurements and from the acclimation period to the end of the study. After an acclimation period (until BP, HR, and MSNA became stable; up to 15 min), baseline beat‐by‐beat BP, HR, and MSNA data were recorded for 5 min. All subjects then performed the isometric HG exercise at 30% of MVC until fatigued, thus when the volunteer could no longer maintain the desired force, coupled with subjective reporting. Blood samples were collected at baseline and at the peak of the HG exercise. To ensure that blood samples were obtained at the exercise peak, all subjects were asked to foretell fatigue at least 30 s before they stopped exercising. The blood samples were centrifuged, and the serum and plasma samples were stored at −80°C prior to analysis. Serum FABP4 concentrations were measured using an enzyme‐linked immunosorbent assay kit according to the manufacturer's protocol (Biovendor R&D, Brno, Czech Republic, Cat. No. RD191036200R). As specified by the manufacturer, the lower limit of detection of serum FABP4 was 0.05 ng/mL, the intra‐assay coefficient of variation was 2.7%. The plasma concentrations of NE, glucose, glycosylated A1a‐1 hemoglobin (HbA1c), triacylglycerols (TG) and creatinine were measured via high performance liquid chromatography at SRL. Inc. (Tokyo, Japan). An index of renal function, the estimated glomerular filtration rate (eGFR), was calculated using an equation appropriate for Japanese subjects: eGFR (mL/min/1.73 m^2^) = 194 × Cr^−1.094^ × age^−0.287^ × 0.739 (if female) (Matsuo et al., 2009). The body mass index (BMI) was the body weight (kg) divided by the square of the height (m^2^).

## 3 STATISTICAL ANALYSIS

All values are presented as means ± standard deviations (SDs). All statistical analyses were performed with the aid of SPSS software (version 25, IBM Corp., Armonk, NY, USA). BP, HR, MSNA, and blood parameters (FABP4 and NE levels) at baseline and the exercise peak (last 30 s of the HG exercise) were compared using the paired *t*‐test. Univariate linear regression analyses were performed to determine the association between the change in absolute value in serum FABP4 levels and the changes in other parameters (MSNA, NE level) during exercise. A two‐sided *p*‐value < 0.05 was considered significant.

## 4 RESULTS

The baseline characteristics of all participants are shown in Table 1. No subject was obese (average BMI 21.7 ± 3.0 kg/m^2^). The BP, HR, and metabolic parameters were within normal ranges (Table 1). MSNA at rest and during exercise were successfully recorded from 14 subjects (Table 1).

**FIGURE 1:**
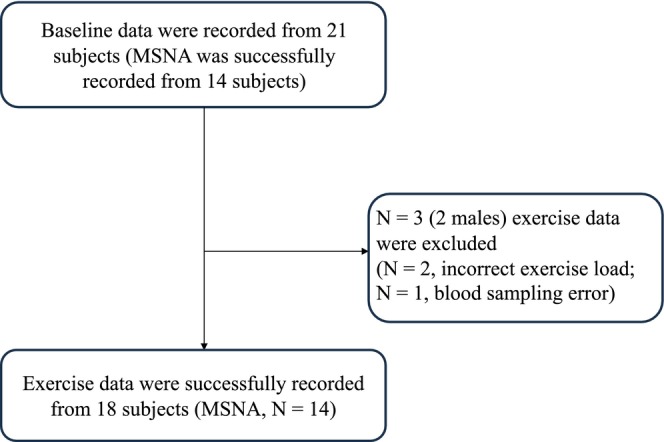
A subject flow diagram. Baseline data were recorded from 21 subjects. MSNA data were successfully obtained from 14 of these individuals.

Exercise data on three subjects, including two men (average age, 28.7 ± 1.7 years; average BMI 21.3 ± 3.5 kg/m^2^), were excluded because the exercise was not correctly performed (e.g., stopped before fatigue because of the pain in fingers), or blood sampling process was problematic (e.g., coagulation). The resting data of these subjects were included (Figure 1).

**FIGURE 2:**
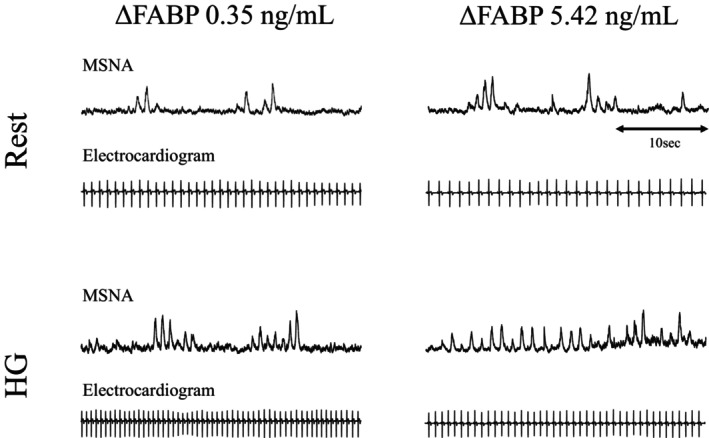
Typical recordings of MSNA. At rest and during exercise in a participant with a large MSNA and FABP4 increase versus in a participant with a small MSNA and FABP4 increase. The magnitude of change in FABP4 is positively correlated with the rate of increase in MSNA.

**FIGURE 3:**
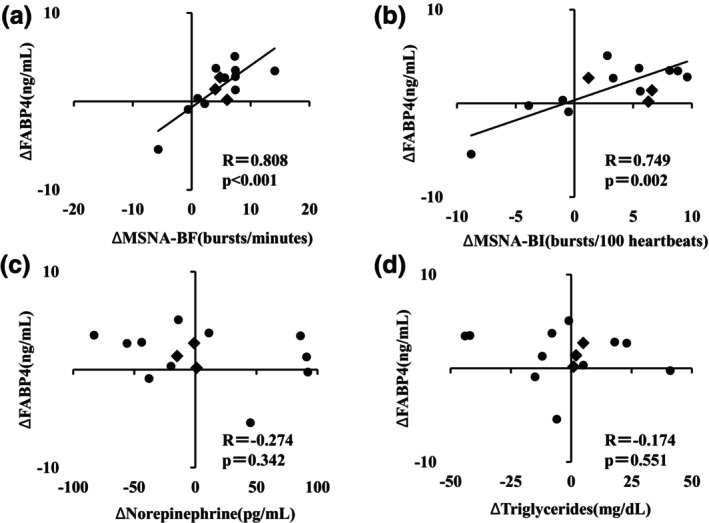
Relationships between the change in FABP4 by HG exercise and other parameters. The axes of the figure represent the change in FABP4, MSNA, NE, or TG. The changes in FABP4 were significantly correlated with the variation in MSNA (a, b) but not with changes in NE (c) or TG (d) levels. Male participants are represented by black circles, while female participants are represented by diamond symbols.

The average MVC was 32.2 ± 4.1 kg, and the average exercise duration was 4 min 11 ± 32 s. As expected, all of the SBP, HR, and MSNA were significantly elevated by HG exercise (all *p* < 0.05) (Table 2). The serum FABP4 concentration was also significantly increased (*p* < 0.05) (Table 2) but the serum concentration of NE did not change (*p* = 0.76) (Table 2). The results of univariate regression between the FABP4 levels and other parameters at rest are shown in Table 3. Typical recordings of MSNA are shown in Figure 2. The FABP4 concentration was not correlated with any parameter, including the serum NE concentration and MSNA (Table 3). The change in FABP4 on exercise was significantly correlated with the change in MSNA BF (bursts/min) (*R* = 0.808, *p* < 0.001) (Figure 3a) and the MSNA BI (bursts/100 heartbeats) (*R* = 0.749, *p* = 0.002) (Figure 3b). The changes in NE (pg/mL) and TG (mg/dL) levels did not correlate with the change in FABP4 concentration (ng/mL) (NE, *R* = −0.274, *p* = 0.342; TG, *R* = −0.174, *p* = 0.551) (Figure 3c,d, respectively).

**TABLE 2:** The heart rate, blood pressure, FABP4, norepinephrine, and sympathetic nerve responses to handgrip exercise.

	Baseline			Handgrip exercise			*p‐*value
Heart rate (beats/min)	64	±	9	78	±	18	0.0001*
Systolic blood pressure (mmHg)	118	±	10	126	±	11	0.004*
Diastolic blood pressure (mmHg)	70	±	9	74	±	13	0.04*
Mean atrial pressure (mmHg)	86	±	9	91	±	12	0.004*
FABP4 (ng/mL)	14.8	±	5.7	16.2	±	5.8	0.03*
Norepinephrine (pg/mL)	178.9	±	67.9	182.8	±	84.4	0.76
MSNA (bursts/min)	17.5	±	10.1	22.1	±	10.3	0.002*
MSNA (bursts/100 heartbeats)	27.0	±	15.5	30.1	±	14.1	0.04*

*Note*: The values are means ± SDs.

* *p* < 0.05 compared to baseline.

**TABLE 3:** Regression analysis between FABP4 levels and clinical parameters.

FABP4 at rest	*R*	*p*
Body mass index (kg/m^2^)	0.218	0.343
Heart rate (beats/min)	0.111	0.633
Systolic blood pressure (mmHg)	−0.307	0.175
Creatinine (mg/dL)	−0.049	0.833
Glucose (mg/dL)	−0.130	0.573
Insulin (μU/mL)	0.011	0.963
HbA1c (%)	0.208	0.364
Triglycerides (mg/dL)	0.006	0.981
Norepinephrine (pg/mL)	0.112	0.629
MSNA (bursts/min)	0.303	0.292
MSNA (bursts/100 heart beats)	0.363	0.202

Abbreviations: FABP4, fatty acid binding protein 4; HbA1c, glycosylated A1a‐1 hemoglobin; MSNA, muscle sympathetic nerve activity.

## 5 DISCUSSION

This is the first report to examine the relationship between MSNA and serum concentration of FABP4 at rest and during the isometric HG exercise. The novel findings of the present study are as follows. (1) Different from our hypothesis, serum FABP4 level at rest was not associated with any parameters including MSNA, TG, and NE in healthy individuals. (2) BP, HR, serum FABP4, and MSNA were significantly increased by HG exercise. The change in FABP4 by HG exercise was significantly correlated with the change in MSNA but not correlated with other parameters including serum NE.

### 5.1 Resting FABP4 level

FABP4 is an abundantly expressed 14–15‐kDa protein that reversibly binds hydrophobic ligands such as long‐chain fatty acids and other lipids (Cao et al., 2013). FABP4 is released into the circulation from adipocytes during lipid breakdown, together with non‐esterified fatty acids and glycerol. Therefore, it seemed plausible that serum FABP4 levels would be associated with lipid/metabolic profiles. Indeed, in prior reports, the serum FABP4 level was significantly correlated with both the BMI and the serum TG level (Xu et al., 2006). The relationships with the lipid/metabolic profiles aside, the resting serum FABP4 level has been associated with cardiovascular risks and diseases (e.g., age, BP, and coronary artery disease) (Chen et al., 2017; Hansen et al., 2021; Kralisch & Fasshauer, 2013; Obokata et al., 2018; Wang et al., 2019). However, unlike previous reports, in the present study the resting serum FABP4 level was not associated with any parameter, including MSNA, possibly because the participant characteristics differed. Prior studies included patients with CVDs and/or metabolic syndrome (Chen et al., 2017; Hansen et al., 2021; Kralisch & Fasshauer, 2013; Obokata et al., 2018; Wang et al., 2019). We enrolled only young healthy individuals who were non‐obese and non‐hypertensive, and their lipid profiles were normal. The data distributions might thus have been too narrow (i.e., within the normal ranges) to detect significant relationships. A significant relationship between SNA and metabolic rate in healthy humans was reported (Day et al., 2005; Monroe et al., 2001), and the degree of sympathetic support of metabolic rate was varied by age, lifestyle, and sex (Bell et al., 2001). Therefore, it looks possible that the association of MSNA with FABP4 is also varied by population. To clarify the relationship between FABP4 and MSNA at rest in more detail, future studies including various populations (e.g., both healthy controls and patients with CVD) are warranted.

### 5.2 Changes in serum FABP4 levels with exercise

As expected, BP, HR, and MSNA were all significantly increased by HG exercise. but only the change in the MSNA significantly correlated with the variation in FABP4 level; the BP and HR changes did not. Hemodynamic changes during exercise are regulated not only by SNA but also by other factors. Parasympathetic withdrawal, sympathetic transduction (O'Brien et al., 2021), and local metabolites (Venturelli et al., 2022) can change the hemodynamics. Therefore, it seems reasonable that only the change in MSNA, a parameter reflecting the efferent sympathetic nerve activities of vascular beds, correlated with the variation in FABP4, supporting our hypothesis that the FABP4 level reflects efferent SNA. In a previous study, unlike what we found, the serum NE level increased with exercise, and the change correlated significantly with the FABP4 variation (Iso et al., 2017). As described in the “Introduction”, use of the serum NE level as a marker of efferent SNA is associated with several limitations including NE release and re‐uptake (i.e., clearance), and the non‐organ‐specific characteristics thereof (i.e., the sum of all NE spilled by various organs, such as from heart and kidney, is measured) (Esler et al., 1990; Mayer et al., 2006). These limitations may explain the lack of a significant relationship between the changes in FABP4 and NE levels during exercise in the present study. Another possible explanation is that the types of exercise differed. In a previous study (Iso et al., 2017), strong loads that attained the anaerobic threshold were applied using an ergometer, whereas our study employed the relatively weaker loads of HG exercise; the hemodynamic response was clearly smaller than in the previous study. It seems possible that the exercise load was too weak to dynamically affect serum NE values. In this study, MSNA, an established measure of efferent SNA, recording detected a significant correlation between MSNA and FABP4 levels, although the change was rather small. This implies that the FABP4 concentration reflects even small variations in SNA induced by HG exercise, and might thus be a more sensitive marker of SNA than the serum NE level.

### 5.3 Perspective

Elevated SNA reflects the severity of certain diseases including sleep apnea syndrome (Hamaoka et al., 2020) and heart failure (Ikeda et al., 2012). Several neuromodulation therapies targeting autonomic nerve dysfunction have been developed (Biffi et al., 2023; Zannad et al., 2015). However, in clinical practice, there are no established markers that can accurately reflect sympathetic activity, which may decelerate the development of neuromodulation therapies. Although the significance of individual differences in FABP4 levels remains unknown, our findings suggest that the rapid changes in FABP4 concentration may partly reflect changes in sympathetic outflow in individuals. Therefore, monitoring FABP4 levels might be a helpful way to track acute changes in SNA brought on by shifts in disease severity or the effects of treatment. Nevertheless, more research including patients with abnormal autonomic function at rest is needed to determine the potential of FABP4 as a marker of SNA.

### 5.4 Limitations

Our study had certain limitations. First, all participants were young and healthy; the findings cannot be generalized to patients with CVD, who are likely to be older and overweight. Second, all data were obtained only at rest and when MSNA was increased by HG. Future studies evaluating FABP4 concentrations under various conditions, such as the change in the relationship through diurnal variation are required. Third, maximum fatigue of participants was self‐declared and may not be objectively guaranteed. However detailed instruction and practice opportunity were provided before the recording, it is possible that some subjects could not reach their actual fatigue point, or the procedure was not ideal (e.g., not static or also contracting other muscle groups). Meanwhile, we found a significant linear relationship between MSNA and FABP4, indicating that when the sympathetic response was low, there was also a small change in FABP4. Therefore, we believe that the possibility of some subjects exercising at a lower intensity does not compromise our conclusion. Fourth, the sample size was relatively small. There is the possibility that the obtained results were underpowered given the low sample size. Yet it should be noted that when power analysis was performed referencing the prior report using the similar study design, which showed the significant linear relationship between FABP4 and serum NE (Iso et al., 2017), the required sample size was calculated to be 11 cases which was smaller than our sample size (assuming a two‐tailed test for the population correlation coefficient with an expected correlation coefficient of 0.7, a significance level of 5%, and a power of 80%) (G*Power 3.1.9.7) (Faul et al., 2009). Fifth, we excluded total MSNA from the analyses. In some subjects, however, the MSNA bursts were clearly countable, burst area was compromised by the change in baseline noise by exercise including the mix of muscle contraction signal (i.e., electromyography), resulting in decreased number of reliable data. Therefore, we decided to exclude the total MSNA data from this study. Lastly, given the relatively small sample size, we cannot evaluate any possible difference between the sexes. It has been recognized that hormonal change through menstrual cycle could affect hemodynamics (Joyner et al., 2016; Shoemaker et al., 2001; Yang et al., 2012). Also, the adipose tissue volume of females differs from that of males (Hu et al., 2016), and androgen may affect serum FABP4 levels (Karastergiou et al., 2012). Therefore, evaluating the sex differences in the relationship between SNA and FABP4 is crucial. Future larger‐scale studies are needed for clarify this issue.

## 6 CONCLUSION

In conclusion, in this study, serum FABP4 levels at rest were not associated with MSNA. However, changes in serum FABP4 levels induced by HG exercise were significantly correlated with the change in MSNA, implying that the serum FABP4 level may, in part, reflect efferent sympathetic drive. Further studies with a broader population including individuals with elevated resting SNA are required to investigate the relationships in more detail.

## AUTHOR CONTRIBUTIONS

*Conceptualization*: Hirai T, Hamaoka T, Murai H, Sugimoto H, Ikeda T. *Data curation*: Hirai T. *Formal analysis*: Hirai T, Ikeda T. *Investigation*: Hirai T, Murai H, Sugimoto H, Mukai Y, Nomura A, Kusayama T, Ikeda T. *Methodology*: Hirai T, Murai H, Sugimoto H, Mukai Y, Nomura A, Ikeda T, Kato T, Usui S. *Project administration*: Hirai T. *Resources*: Murai H, Takashima S, Sakata K, Usui S, Takamura M. *Supervision*: Hamaoka T, Ikeda T, Kato T, Sakata K, Takamura M. *Validation*: Hamaoka T, Ikeda T, Takashima S, Takata S, Takamura M. *Visualization*: Hirai T. *Writing—original draft*: Hirai T. All co‐authors approved the final version of the manuscript.

## FUNDING INFORMATION

This work was partially supported by the Otsuka Medical Devices Co. Ltd.

## CONFLICT OF INTEREST STATEMENT

The authors declare no conflicts of interest.

## ETHICS STATEMENT

The study protocol was approved by the Research Ethics Board of Kanazawa University and conformed with the Declaration of Helsinki. All participants provided written informed consent.

## CONSENT STATEMENT

All subjects gave us written consent for publication.

## Data Availability

The data that support the findings of this study are available from the corresponding author upon reasonable request.
